# Supplementary material for: Intra- and inter-molecular regulation by intrinsically-disordered regions governs PUF protein RNA binding
Source: Nat Commun. 2023 Nov 13;14:7323. doi: 10.1038/s41467-023-43098-1 (PMC10641069; doi:10.1038/s41467-023-43098-1)
Supplement: Supplementary file 2 — Supplementary Information file [file 41467_2023_43098_MOESM2_ESM.pdf]

Intra- and inter-molecular regulation by intrinsically-disordered regions governs PUF protein RNA binding

Supplementary Information

Supplementary Tables

Supplementary Table 1. Melting temperatures of FBF-2 variants measured by differential scanning calorimetry (n=3 independent experiments). Source data are provided as a Source Data file.

**FBF-2**	**T_m (°C)** [1]
RBD	36.32 ± 0.05
RBD+CT	40.24 ± 0.12
RBD+CT L610A	35.63 ± 0.05
RBD+CT Y479A	35.25 ± 0.05
RBD+CT Loop6A	37.58 ± 0.05

[1]Mean ± SD from three technical replicates.

Supplementary Table 2. Crystallographic data collection and refinement statistics.

	**FBF-2 RBD+CT/cFBE**
Resolution range[1] (Å)	50-2.09 (2.13-2.09)
Space group	$P6_1$
Unit cell dimensions<br>a, b, c (Å)<br>α, β, γ (°)	93.4, 93.4, 110.5<br>90, 90, 120
Unique reflections[2]	32508 (1636)
Multiplicity	8.7 (7.5)
Completeness (%)	99.9 (100)
Mean I/sigma(I)	18 (1.7)
Wilson B-factor	35.6
R-meas	0.15 (1.61)
R-pim	0.05 (0.58)
**Refinement**	
Reflections used in refinement	32350
Reflections used for R-free	1990
R-work	0.165 (0.238)
R-free	0.205 (0.268)
Number of atoms	
protein	3244
RNA	190
solvent	181
RMSD bonds (Å)	0.005
RMSD angles (°)	0.66
Ramachandran favoured (%)	98.99
Ramachandran outliers (%)	0
Average B-factors ($Å^2$)	
protein	43.6
RNA	56.2
solvent	46.4

[1]The highest-resolution shell is shown in parentheses.

[2]Statistics for the highest-resolution shell are shown in parentheses.

Supplementary Table 3. Protein interaction affinities, stoichiometries, and thermodynamic parameters between FBF-2 CT/LST-1 variants and FBF-2 measured by ITC. Two technical replicates (n=2 independent experiments) were performed for each analysis with similar results, and the values are shown for each replicate. Source data are provided as a Source Data file.

FBF-2	Peptide	RNA	K_d (μM)	N (sites)	ΔH (kJ/mol)	ΔG (kJ/mol)	-TΔS (kJ/mol)
**RBD**	FBF-2 CT (601-632)	none	36.2<br>34.3	0.80<br>1.00	-41.3<br>-40.3	-25.0<br>-25.1	16.4<br>15.2
**RBD**	LST-1 B (67-98)[1]	none	0.046<br>0.045	0.87<br>0.88	-73.4<br>-71.3	-41.2<br>-41.3	32.2<br>30.0
**RBD**	LST-1 A (19-50)[1]	none	2.13<br>2.02	0.88<br>0.85	-57.3<br>-57.1	-31.9<br>-32.0	25.4<br>25.1
**RBD+CT**	LST-1 B (67-98)	none	2.1<br>3.3	0.82<br>0.83	-40.6<br>-40.2	-31.9<br>-30.8	8.7<br>9.5
**RBD+CT**	LST-1 A (19-50)	none	Not detected				
**RBD**	FBF-2 CT L610A (601-632)	none	Not detected				
**RBD+CT L610A**	LST-1 B (67-98)	none	0.077<br>0.046	0.85<br>0.73	-65.0<br>-70.5	-40.0<br>-41.2	25.1<br>29.2
**RBD+CT L610A**	LST-1 A (19-50)	none	2.6<br>2.1	0.83<br>0.84	-50.0<br>-51.7	-31.4<br>-31.9	18.6<br>19.8
**RBD+CT Loop6A**	LST-1 B (67-98)	none	1.1<br>1.3	1.06<br>0.99	-54.8<br>-48.1	-33.5<br>-33.1	21.2<br>15.0

[1] Reported previously [18].

Supplementary Table 4. RNA-binding affinities measured by EMSA. Source data are provided as a Source Data file.

**FBF-2**	**RNA**	**LST-1**	**K_d (nM)**[1]	**n**[2]
RBD	FBEa	none	70 ± 23	4
RBD	FBEa	67-98 (B)	176 ± 27	3
RBD+CT	FBEa	none	334 ± 61	4
RBD+CT, L610A	FBEa	none	51 ± 26	5
RBD+CT	FBEa	19-50 (A)	114 ± 10	3
RBD+CT	FBEa	67-98 (B)	68 ± 21	3
RBD+CT, L610A	FBEa	67-98 (B)	82 ± 18	3
RBD+CT, Y479A	FBEa	none	62 ± 14	3
RBD+CT, Y479A	FBEa	67-98 (B)	41 ± 6	3
RBD+CT, Loop6A	FBEa	none	101 ± 21	3
RBD+CT, Loop6A	FBEa	67-98 (B)	166 ± 21	3

[1]Mean ± SEM from indicated number of independent technical replicates.

[2]n=number of independent technical replicates with similar results.

Supplementary Table 5. Hydrogen bond occupancies.

**Acceptor residue (atom)**	**Donor residue (atom)**	**% Occupancy[1]**
U2 (O4)	His593 (H)	36.0
Glu 592 (OE2)	U2 (H3)	9.8
Glu 592 (OE1)	U2 (H3)	7.0
U2 (O2)	Asn 601 (HD21)	6.1
U2 (O2)	Asn 601 (HD22)	5.0
Ser 591 (O)	A3 (H61)	19.7
A3 (N7)	Ser 588 (HG)	6.9
Phe 495 (O)	U4 (H3)	23.9
U4 (O2)	Gln 497 (H)	9.8
U4 (OP2)	Arg 551 (HH22)	8.2
U4 (OP2)	Arg 551 (HH12)	5.9

[1]Occupancies less than 5% are not included.

Supplementary Table 6. Molecular Dynamics simulations setup.

**Parameter**	
Simulation box dimensions	14 nm x 13 nm x 13 nm
Total number of atoms	7,503
Total number of water molecules	66,064
Salt concentration	150 mM NaCl (381 total salt ions with 200 Na^+ and 181 Cl^-)
Lipid composition	N/A

Supplementary Figures

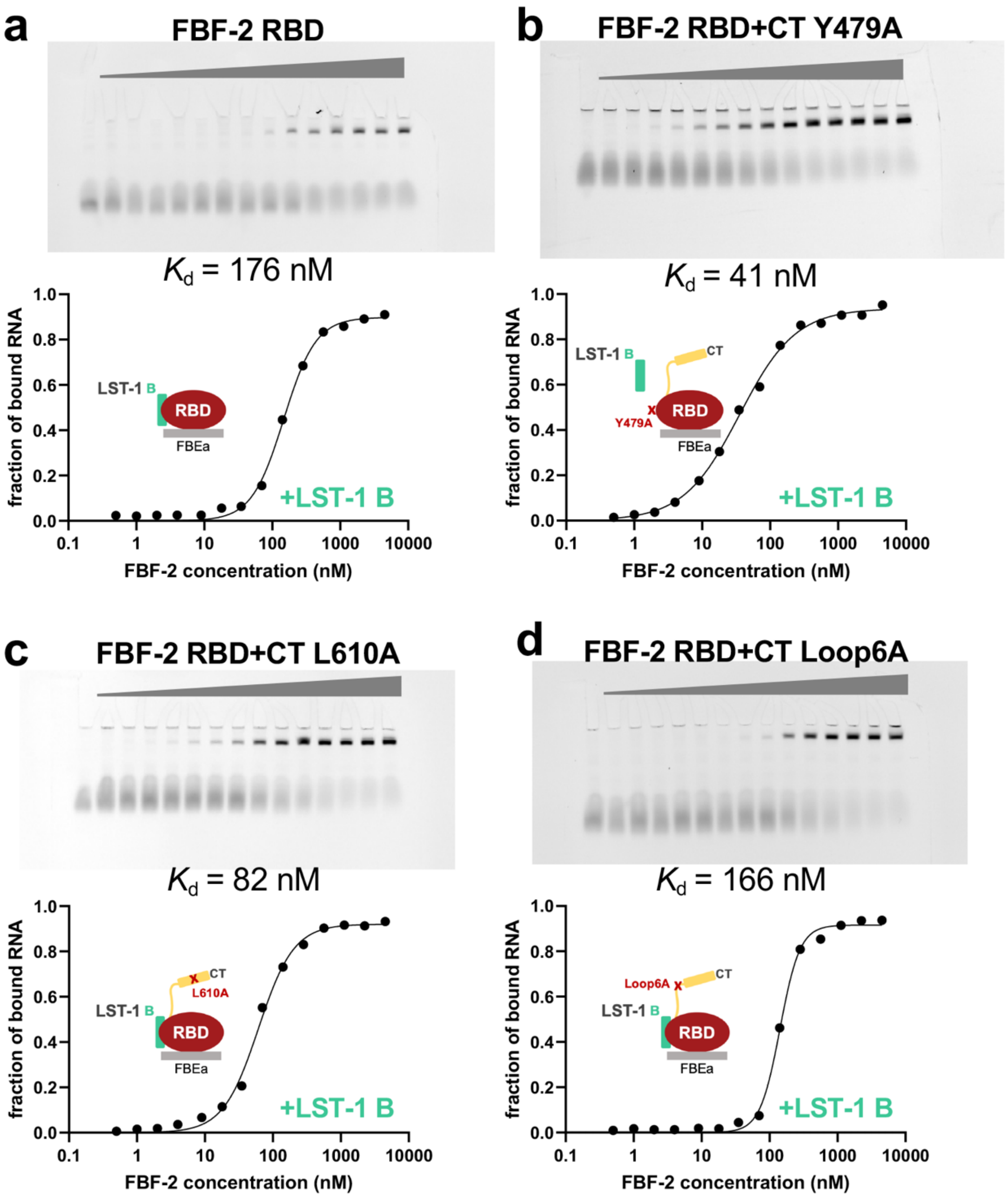

Supplementary Figure 1. Effects of LST-1 B on RNA-binding affinity of FBF-2. Representative EMSA gels (uncropped and unaltered) and binding curves are shown for binding to FBEa RNA in the presence of LST-1 B by (**a**) FBF-2 RBD, (**b**) FBF-2 RBD+CT Y479A, (**c**) FBF-2 RBD+CT L610A, and (**d**) FBF-2 RBD+CT Loop6A. The left lanes are RNA only. Data points on the graphs are shown as filled circles. Mean K_d from at least three independent technical replicates with similar results are indicated (FBF-2 RBD, n=3 independent experiments; FBF-2 RBD+CT Y479A, n=3; FBF-2 RBD+CT L610A, n=3; and FBF-2 RBD+CT Loop6A, n=3.) See also Supplementary Table 4. Source data are provided as a Source Data file.

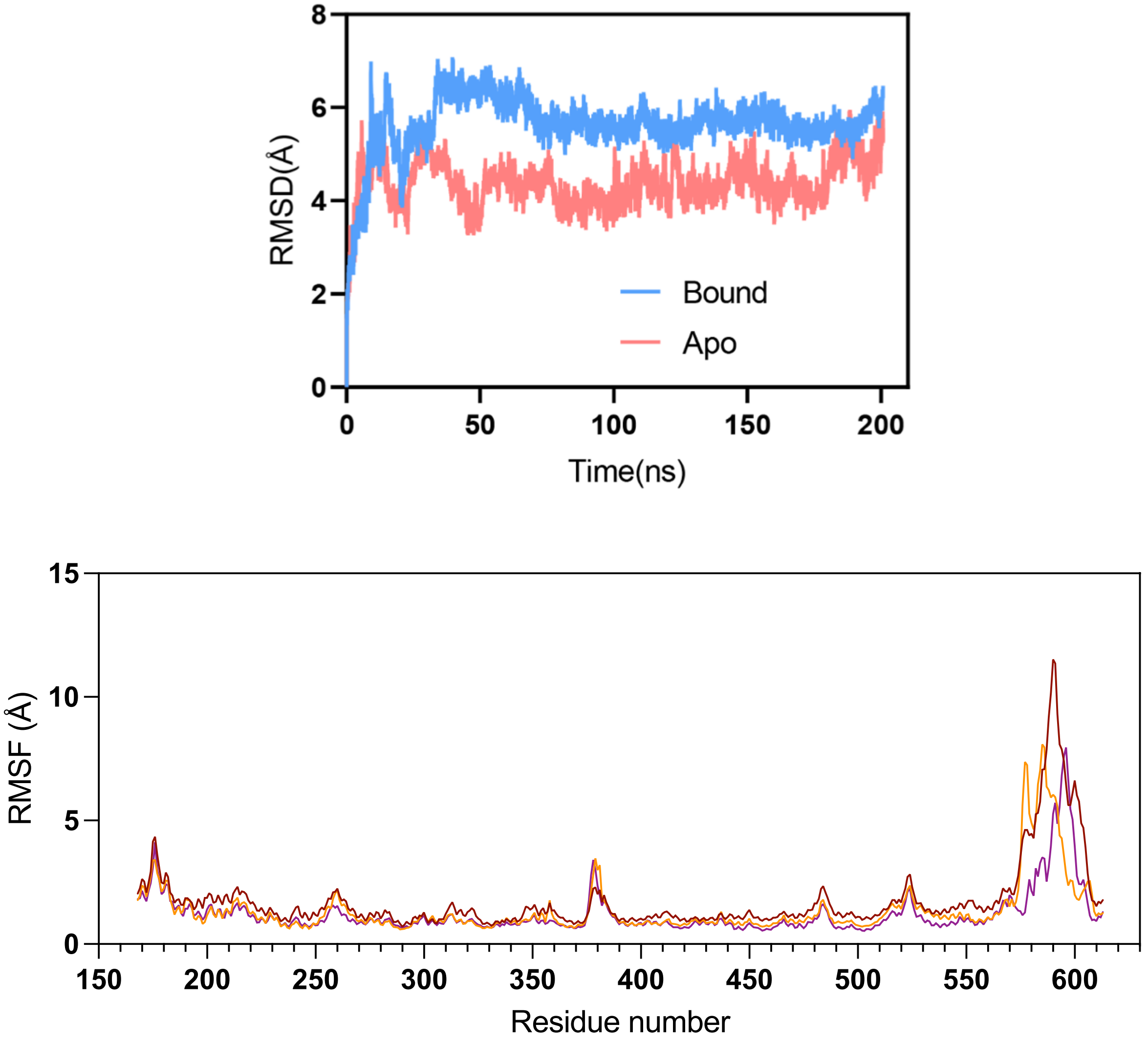

Supplementary Figure 2. RMSD for CA atoms over the course of MD simulations for models of FBF-2 RBD+CT bound to RNA (blue) or apo (red) of representative runs (Top panel). The RMSF analysis for three independent runs are shown in the bottom panel.

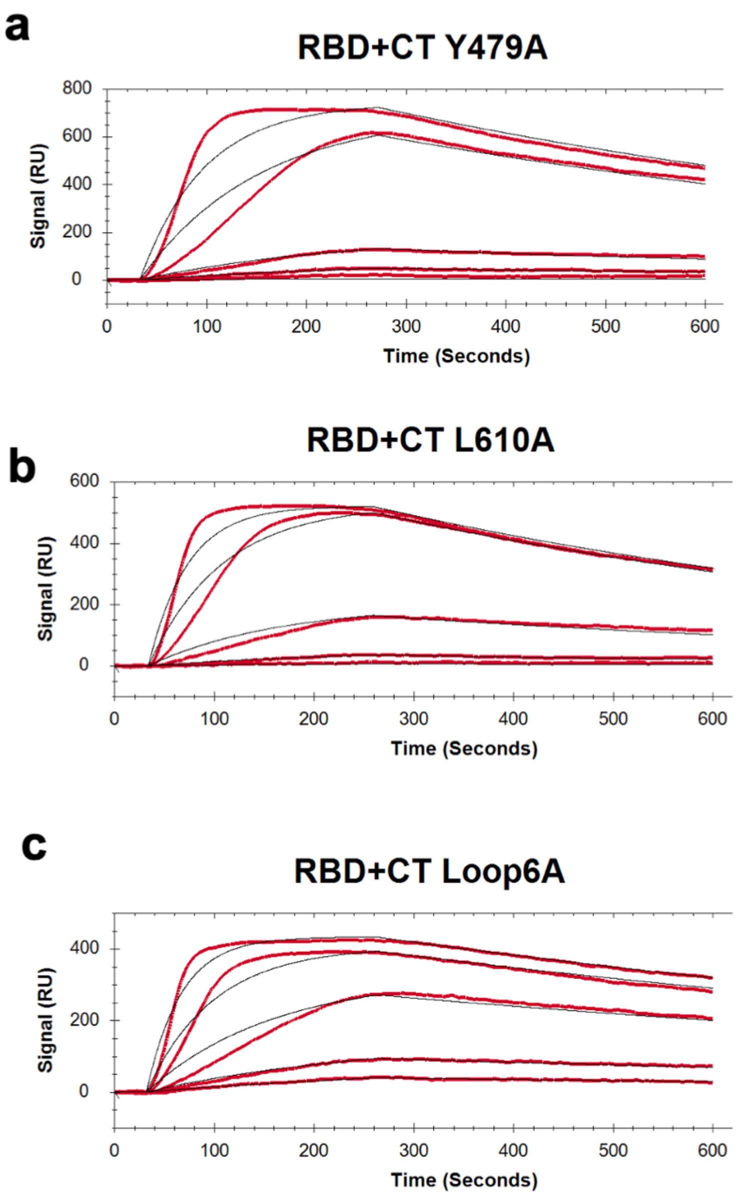

Supplementary Figure 3. Representative SPR binding curves (red) and fitted curves (black) for FBF-2 RBD+CT mutants (**a**) Y479A, (**b**) L610A, and (**c**) Loop6A. Five protein concentrations were run shown from top to bottom: 200, 100, 50, 25, and 12.5 nM. See also Figure 5c. Two technical replicates (n=2 independent experiments) were performed with similar results. Source data are provided as a Source Data file.

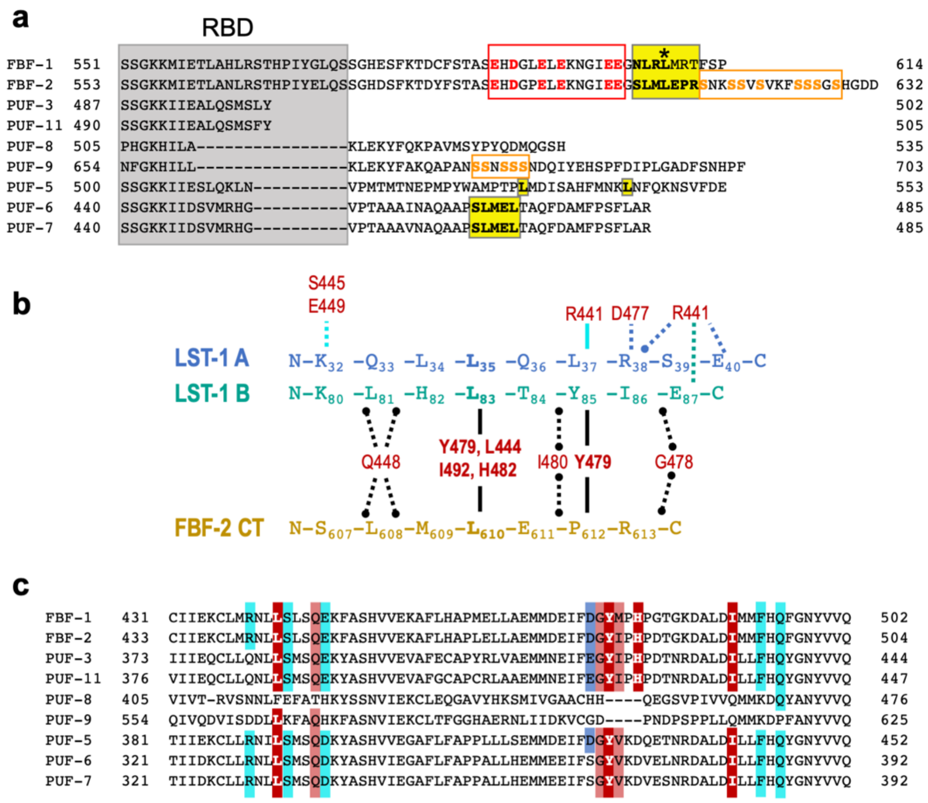

d

PUF protein	PIM binding residues	CT PIM	Acidic Cluster	Serine Cluster
FBF-1	**yes**	maybe	**yes**	no
FBF-2	**yes**	**yes**	**yes**	**yes**
PUF-3, PUF-11	**yes**	no	no	no
PUF-8	no	no	no	no
PUF-9	no	no	no	**yes**
PUF-5	**yes**	maybe	no	no
PUF-6, PUF-7	**yes**	maybe	no	no

Supplementary Figure 4. Comparison of *C. elegans* PUF proteins. (**a**) Amino acid sequence alignment of *C. elegans* PUF protein CTs. Residues at the end of the RBD are boxed in grey. The FBF-2 CT PIM and possible PIMs in other PUF proteins are highlighted yellow. The FBF-2 electronegative cluster is shown in red, and serine clusters are shown in orange. (**b**) Schematic drawing of interactions of FBF-2 RBD with CT, LST-1 A, or LST-1 B peptides. FBF-2 interactions with all three peptides (middle, black), with both A and B peptides (top, cyan), specific to LST-1 A (top, blue), or specific to LST-1 B (top, green) are indicated by dashed lines. Interactions via main chain atoms end with circles. (**c**) Amino acid sequence alignment of *C. elegans* PUF protein PIM interacting residues. Residues that form the hydrophobic pocket around the critical leucine residues are highlighted red with white letters. Residues that contact all three peptides (light red), both LST-1 A and B peptides (cyan), or specific to LST-1 A (blue) are also highlighted. (**d**) Table summarizing potential regulatory features in *C. elegans* PUF proteins.
