# Supplementary material for: Intra- and inter-molecular regulation by intrinsically-disordered regions governs PUF protein RNA binding
Source: Nat Commun. 2023 Nov 13;14:7323. doi: 10.1038/s41467-023-43098-1 (PMC10641069; doi:10.1038/s41467-023-43098-1)
Supplement: Supplementary file 3 — Description of additional supplementary files [file 41467_2023_43098_MOESM3_ESM.pdf]

Description of additional supplementary files

Supplementary Data 1 - Starting coordinates for the molecular dynamics simulation.

Supplementary Data 2 - Ending coordinates for the molecular dynamics simulation.

Supplementary Data 3 – Molecular dynamics initial input file.

Supplementary Data 4 – Molecular dynamics extension input file.

Supplementary Movie 1 - Movie of the molecular dynamics simulation of FBF-2 RBD+CT168-613 in complex with gld-1 FBEa RNA. FBF-2 RBD is red, CT region is yellow, and RNA is cyan. Note the correlated motions of the 5´ end of the RNA and the CT region loop.
