# Supplementary material for: Intra- and inter-molecular regulation by intrinsically-disordered regions governs PUF protein RNA binding
Source: Nat Commun. 2023 Nov 13;14:7323. doi: 10.1038/s41467-023-43098-1 (PMC10641069; doi:10.1038/s41467-023-43098-1)
Supplement: Supplementary file 9 — Reporting Summary [file 41467_2023_43098_MOESM9_ESM.pdf]

Corresponding author(s): Traci Hall

Last updated by author(s): Sep 15, 2023

Reporting Summary

Nature Portfolio wishes to improve the reproducibility of the work that we publish. This form provides structure for consistency and transparency in reporting. For further information on Nature Portfolio policies, see our Editorial Policies and the Editorial Policy Checklist.

Statistics

For all statistical analyses, confirm that the following items are present in the figure legend, table legend, main text, or Methods section.

n/a	Confirmed	
☐	☒	The exact sample size (*n*) for each experimental group/condition, given as a discrete number and unit of measurement
☒	☐	A statement on whether measurements were taken from distinct samples or whether the same sample was measured repeatedly
☒	☐	The statistical test(s) used AND whether they are one- or two-sided<br>*Only common tests should be described solely by name; describe more complex techniques in the Methods section.*
☒	☐	A description of all covariates tested
☒	☐	A description of any assumptions or corrections, such as tests of normality and adjustment for multiple comparisons
☐	☒	A full description of the statistical parameters including central tendency (e.g. means) or other basic estimates (e.g. regression coefficient) AND variation (e.g. standard deviation) or associated estimates of uncertainty (e.g. confidence intervals)
☒	☐	For null hypothesis testing, the test statistic (e.g. *F*, *t*, *r*) with confidence intervals, effect sizes, degrees of freedom and *P* value noted<br>*Give P values as exact values whenever suitable.*
☒	☐	For Bayesian analysis, information on the choice of priors and Markov chain Monte Carlo settings
☒	☐	For hierarchical and complex designs, identification of the appropriate level for tests and full reporting of outcomes
☒	☐	Estimates of effect sizes (e.g. Cohen's *d*, Pearson's *r*), indicating how they were calculated

Our web collection on statistics for biologists contains articles on many of the points above.

Software and code

Policy information about availability of computer code

Data collection	X-ray diffraction data were collected using SERGUI (SER-CAT beamline, APS).<br>ITC data were collected using the MicroCal PEAQ-ITC Automated Control Software (Version 1.40).<br>SPR data were collected using OpenSPR (Nicoya, version 4.3).
Data analysis	Protein Thermal ShiftTM software (Applied Biosystems, version 1.4) was used to analyze protein melting curves and calculate melting temperatures (Tm).<br>X-ray diffraction data sets were scaled with HKL2000 (HKL Research).<br>Molecular replacement was performed with Phaser in Phenix version 1.20-4459.<br>X-ray crystallographic refinement was performed with Phenix version 1.20-4459.<br>Manual model building was performed with Coot (version 0.9.6 EL).<br>ITC data were analyzed using the MicroCal PEAQ-ITC Analysis Software (version 1.22).<br>EMSA band intensities were quantified with ImageQuant 5.2 (Cytiva).<br>EMSA data were fit with GraphPad Prism (version 9.2.0).<br>The Amber20 package with AMBER force fields ff19SB, RNA.OL3 and water.opc was used for MD simulations.<br>For each SPR experiment, binding curves were fit with the TraceDrawer software (Ridgeview Instruments, version 1.9.1).

For manuscripts utilizing custom algorithms or software that are central to the research but not yet described in published literature, software must be made available to editors and reviewers. We strongly encourage code deposition in a community repository (e.g. GitHub). See the Nature Portfolio guidelines for submitting code & software for further information.

Data

Policy information about availability of data

All manuscripts must include a data availability statement. This statement should provide the following information, where applicable:

- Accession codes, unique identifiers, or web links for publicly available datasets
- A description of any restrictions on data availability
- For clinical datasets or third party data, please ensure that the statement adheres to our policy

Atomic coordinates and structure factors for the reported crystal structure have been deposited with the Protein Data Bank under accession number 8SJ7. PDB 3V74 was used as a model for molecular replacement. All other data supporting the findings of this study are available within the paper and its Supplementary Information. Source data are provided with this paper.

Research involving human participants, their data, or biological material

Policy information about studies with human participants or human data. See also policy information about sex, gender (identity/presentation), and sexual orientation and race, ethnicity and racism.

Reporting on sex and gender	Not applicable, no human participants, data, or biological material were used.
Reporting on race, ethnicity, or other socially relevant groupings	Not applicable
Population characteristics	Not applicable
Recruitment	Not applicable
Ethics oversight	Not applicable

Note that full information on the approval of the study protocol must also be provided in the manuscript.

Field-specific reporting

Please select the one below that is the best fit for your research. If you are not sure, read the appropriate sections before making your selection.

☒ Life sciences ☐ Behavioural & social sciences ☐ Ecological, evolutionary & environmental sciences

For a reference copy of the document with all sections, see nature.com/documents/nr-reporting-summary-flat.pdf

Life sciences study design

All studies must disclose on these points even when the disclosure is negative.

Sample size	For in vitro assays: EMSAs were conducted at least in triplicate and values are reported as Kd ± SEM. This is a customary sample size, which provides the power to detect statistically significant differences, if present. ITC was conducted in duplicate due to the high amount of sample required for an individual replicate, and values for both experiments, which are similar, are reported. SPR was conducted in duplicate at 5 protein concentrations and values are reported as mean ± SD. Duplicate SPR experiments were performed at 5 protein concentrations, representing a sample size of 10.
Data exclusions	No data were excluded.
Replication	Technical replicates for EMSAs, ITC, and SPR experiments produced similar results.
Randomization	This section is not applicable to our in vitro studies.
Blinding	We did not blind the data processing from the data collection. Dr. Qiu performed experiments and also processed the data.

Reporting for specific materials, systems and methods

We require information from authors about some types of materials, experimental systems and methods used in many studies. Here, indicate whether each material, system or method listed is relevant to your study. If you are not sure if a list item applies to your research, read the appropriate section before selecting a response.

Materials & experimental systems

n/a	Involved in the study
☒	☐ Antibodies
☒	☐ Eukaryotic cell lines
☒	☐ Palaeontology and archaeology
☒	☐ Animals and other organisms
☒	☐ Clinical data
☒	☐ Dual use research of concern
☒	☐ Plants

Methods

n/a	Involved in the study
☒	☐ ChIP-seq
☒	☐ Flow cytometry
☒	☐ MRI-based neuroimaging
